# Supplementary material for: Mechanisms governing the pioneering and redistribution capabilities of the non-classical pioneer PU.1
Source: Nat Commun. 2020 Jan 21;11:402. doi: 10.1038/s41467-019-13960-2 (PMC6972792; doi:10.1038/s41467-019-13960-2)
Supplement: Supplementary file 7 — Source data [file 41467_2019_13960_MOESM7_ESM.zip › Source_Data/FigureS2/FigureS2B_MotifScanOutput/KmeansCluster9/homerResults/motif3.similar.html]

motif3

## Information for motif3

C
A
G
T
A
T
G
C
G
T
C
A
A
G
T
C
G
C
A
T
C
A
G
T
G
A
T
C
G
T
A
C
A
C
G
T
C
T
A
G
A
G
C
T
  
Reverse Opposite:  

T
C
G
A
A
G
T
C
C
G
T
A
C
A
T
G
C
T
A
G
G
T
C
A
C
G
T
A
T
C
A
G
A
C
G
T
A
T
C
G
G
T
C
A
  

|  |  |
| --- | --- |
| p-value: | 1e-169 |
| log p-value: | -3.892e+02 |
| Information Content per bp: | 1.573 |
| Number of Target Sequences with motif | 332.0 |
| Percentage of Target Sequences with motif | 14.59% |
| Number of Background Sequences with motif | 807.9 |
| Percentage of Background Sequences with motif | 1.75% |
| Average Position of motif in Targets | 94.5 +/- 46.4bp |
| Average Position of motif in Background | 92.3 +/- 67.9bp |
| Strand Bias (log2 ratio + to - strand density) | -0.8 |
| Multiplicity (# of sites on avg that occur together) | 1.05 |
| Motif File: | file (matrix) reverse opposite |

### Similar de novo motifs found

|  |  |  |  |  |  |  |  |
| --- | --- | --- | --- | --- | --- | --- | --- |
| Rank | Match Score | Redundant Motif | P-value | log P-value | % of Targets | % of Background | Motif file |
| 1 | 0.809 | A G T C A G T C G T C A A G T C C G A T A G C T A G T C A G T C A C G T A C T G A C G T A C T G | 1e-76 | -175.162062 | 6.11% | 0.63% | motif file (matrix) |
